# Analysis of IVF/ICSI Outcomes in Endometriosis Patients With Recurrent Implantation Failure: Influence on Cumulative Live Birth Rate

**DOI:** 10.3389/fendo.2021.640288

**Published:** 2021-07-30

**Authors:** Chenyi Zhong, Liusijie Gao, Li Shu, Zhen Hou, Lingbo Cai, Jie Huang, Jiayin Liu, Yundong Mao

**Affiliations:** ^1^State Key Laboratory of Reproductive Medicine, Center of Clinic Reproductive Medicine, The First Affiliated Hospital of Nanjing Medical University/Jiangsu Province Hospital/Jiangsu Women and Children Health Hospital, Nanjing, China; ^2^OB/GYN Department, The Second Affiliated Hospital of Nantong University, Nantong, China

**Keywords:** endometriosis, recurrent implantation failure, infertility, GnRH-a, cumulative live birth rate

## Abstract

**Objective:**

To study the influence of endometriosis activity on the pregnancy outcomes of patients with recurrent implantation failure (RIF) in *in-vitro* fertilization/intra-cytoplasmic sperm injection (IVF/ICSI) cycles. The pregnancy outcomes were compared between RIF patients with endometriosis who received treatment at different occasions to explore the appropriate treatment plan for these patients and to optimize the pregnancy-support strategies.

**Design:**

Ambispective cohort study.

**Methods:**

A total of 330 patients with endometriosis were enrolled from 2008 to 2018 and included 1043 IVF/ICSI cycles. All patients were diagnosed with RIF after IVF/ICSI. Patients were assigned to three subtypes according to different control states of endometriosis, including the untreated, early-treatment, and late-treatment groups. The clinical pregnancy rate, live birth rate, and cumulative live birth rate of endometriosis patients with RIF were the main outcomes; additionally, the fertilization rate, available embryonic rate, and high-quality embryonic rate were also compared.

**Results:**

The early-treatment and late-treatment groups showed higher cumulative live birth rate than the untreated group (early-treated 43.6% *vs*. late-treated 46.3% *vs*. untreated 27.7%, P<0.001), though patients in the two treatment groups had higher rates of adenomyosis and ovarian surgery. The two treatment group showed a better laboratory result than the untreated and especially, the early-treatment group. The untreated group (46.24%) had a lower IVF fertilization rate than the treated group (early-treated [64.40%] and late-treated [60.27%] (P<0.001). In addition, the rates of available embryos and high-quality embryos in the early-treated group were much higher those that in the untreated group (90.30% *vs*. 85.20%, 76.50% *vs*. 64.47%). Kaplan–Meier curve showed that patients in the untreated group needed a mean of 23.126 months to achieve one live birth; whereas those in the treated group needed a comparatively shorter duration (early-treated: 18.479 ± 0.882 months and late-treated: 14.183 ± 1.102 months, respectively).

**Conclusion:**

Endometriosis has a negative influence on IVF/ICSI outcome. The control of endometriosis activity can result in a higher cumulative live birth rate in patients. It is necessary for endometriosis patients to receive medical treatment to achieve a better prognosis especially for those with RIF.

## Introduction

Infertility is a growing medical concern. With the effects of environmental pollution, social pressure, and changes in the notion of fertility, many couples suffer from infertility. Worldwide, infertility is estimated to affect about 8–12% of couples in the reproductive age (1). Although assisted reproductive technologies (ARTs) have made great progress, sterility is still a big challenge not only for infertile families but also for gynecologists and embryologists.

Endometriosis is an estrogen-dependent, chronic, and aseptic inflammation. The incidence of endometriosis in women of childbearing age is about 10%, and the incidence in infertile women is up to 50% (2, 3). Thus, endometriosis is deemed as an important factor in fertility. The mechanisms of endometriosis-associated infertility are not clear, and the most accepted current theories include the anatomical abnormalities caused by adhesion and fibrosis, endocrine abnormalities, and inflammatory and immune disorders (4). Despite the wide use of ART by endometriosis patients, endometriosis is highly associated with poor outcomes in *in-vitro* fertilization/intra-cytoplasmic sperm injection (IVF/ICSI). Studies (5–7) have found that patients with endometriosis have lower clinical pregnancy rate, poorer ovarian response, lower egg retrieval rate, and higher gonadotropin demand than those with tubal infertility.

The key to successful IVF/ICSI is embryo implantation. However, the average success rate of implantation is about 20% (8) in the common population, and recurrent implantation failure (RIF) continues to be a challenge in ART. RIF is associated with huge economic burden and psychological pressure in patients. In China, the incidence of RIF is 5–11.1% (9). The definition of RIF is controversial (10–13). We reviewed the relevant literature and proposed and adopted the following definition in our study: a condition wherein women aged under 40 years cannot achieve a viable pregnancy after at least three fresh or frozen embryo transfer cycles with more than four high-quality embryos or two high-quality blastocysts transferred (11, 12). Embryonic developmental defects and endometrial receptivity reduction are the two most common reasons leading to RIF (14, 15). Uterine disorders and endocrine abnormality are also said to cause RIF (16–18).

An increasing number of researchers have begun to focus on endometriosis or RIF, but studies on endometriosis-related outcomes in patients with RIF are still limited. Therefore, this study used an ambispective cohort design and grouped RIF patients with endometriosis into the untreated, the early-treatment, and the late-treatment groups based on the different control states of endometriosis to study its effect on pregnancy outcomes. It is important to investigate whether endometriosis affects the pregnancy outcomes of RIF patients and optimize strategies to achieve better pregnancy outcomes.

## Patients and Methods

### Study Design

**Figure 1:**
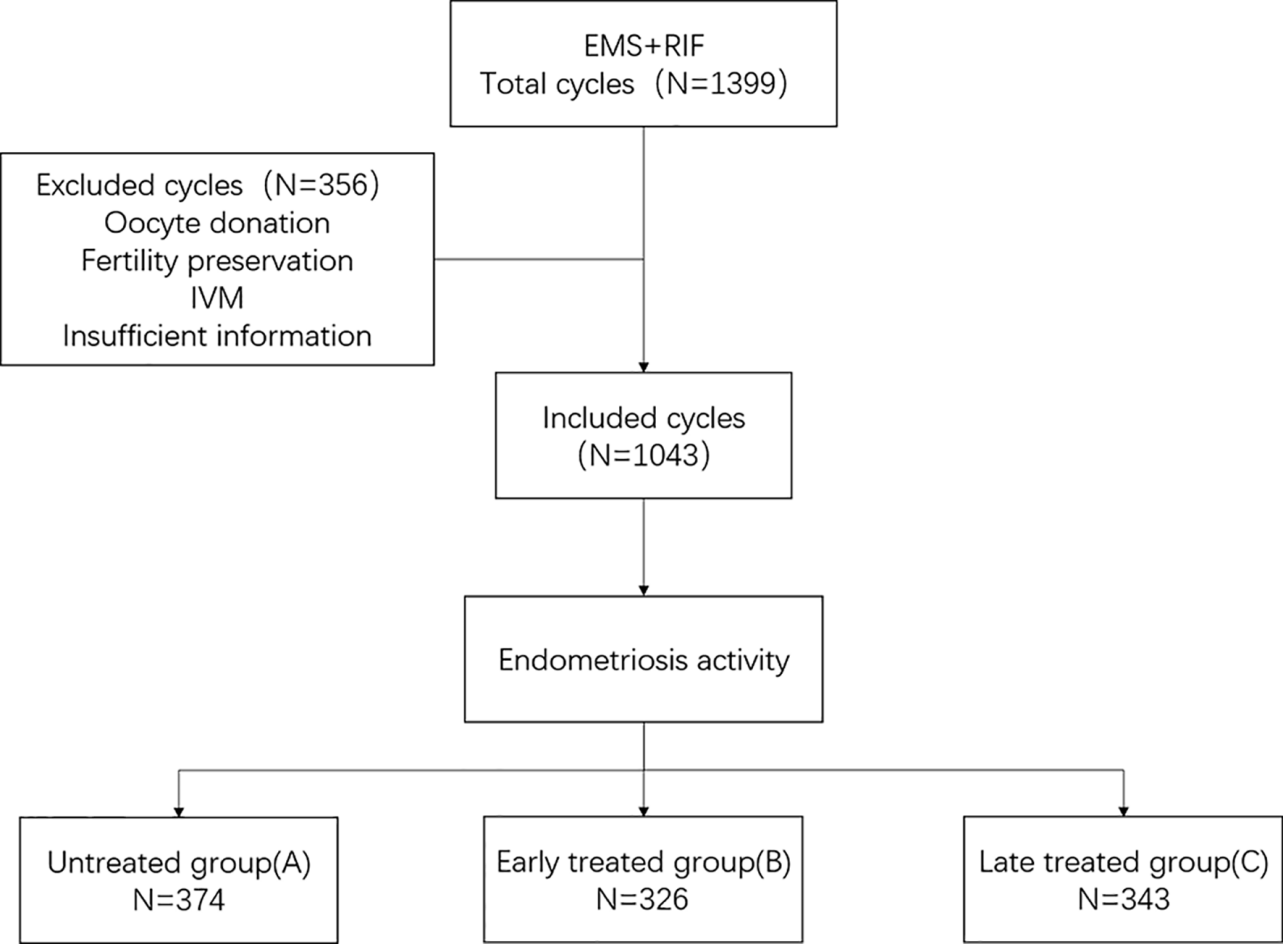
Flowchart of study recruitment and inclusion/exclusion criteria.

We performed an ambispective cohort study by using the clinical assisted reproductive technologies management system software (CCRM) database from the First Affiliated Hospital of Nanjing Medical University from July 2008 to August 2018. All patients with endometriosis and a diagnosis of RIF were considered for inclusion. The exclusion criteria were as follows: Patients (1) receiving oocyte donation, (2) undergoing fertility preservation cycles because of malignant tumors or chemotherapy, (3) undergoing *in vitro* maturation (IVM) cycles, and (4) with incomplete data (**Figure 1**).

Patients were assigned to three subtypes according to different control states of endometriosis. Clinical outcomes including clinical pregnancy rate, live birth rate, and cumulative live birth rate, and laboratory outcomes including the fertilization rate, available embryonic rate, and high-quality embryonic rate were compared. Patients were divided into three groups: Group A (untreated), RIF patients who did not receive treatment for endometriosis; Group B (early treatment), endometriosis patients who received treatments before RIF diagnosis but continued to suffer from RIF; and Group C (late treatment), patients who underwent endometriosis treatments after being diagnosed with RIF (**Supplemental Figure 1**). The treatments for endometriosis were laparoscopic surgery and/or at least two doses of gonadotropin-releasing hormone agonist (GnRH-a).

### Definition of RIF and Endometriosis

In this study, RIF was defined as failure to achieve clinical pregnancy with at least three transfer (fresh embryo or frozen embryo) cycles and cumulatively no less than four high-quality embryos or two high-quality blastocysts.

Endometriosis was defined based on at least one of the following conditions:

1. Laparoscopic or transabdominal surgery confirmed endometriosis;
2. Transvaginal ultrasound showed endometrioma. The “typical” endometrioma manifests as a unilocular or multilocular (less than five locules) cyst with ground glass echogenicity of the cyst fluid (19);
3. Suspected endometriosis: Infertility, dysmenorrhea, dyspareunia, sacral ligament tenderness, and CA125>15 mIU/mL (20). Highly suspected endometriosis can be diagnosed based on any three of the five items (the confirmed diagnosis rate of suspected endometriosis after laparoscopy is 94% in our center).

### Main Outcomes

#### Clinical Outcomes

Cancellation rate/cycle = number of cancelled cycles (including oocyte retrieval failure, unfertilized, not transferred because of poor oocyte quality)/total number of cycles.

Clinical pregnancy rate/ET = number of cycles with clinical pregnancy/number of transfer cycles (clinical pregnancy is defined as ultrasound confirmed gestational sac and/or germ and/or fetal heartbeat).

Early abortion rate = number of early abortion cycles (cycles in which spontaneous abortion occurred before 12 weeks of gestation)/number of clinical pregnancy cycles.

Live birth rate = number of cycles with live births (live births defined as any infant born live after 28 weeks of gestation)/number of transfer cycles.

Cumulative live birth rate = number of cycles in which patients first deliver babies/number of cycles that initiate stimulation.

#### Laboratory Outcomes

Oocyte retrieval rate = number of retrieved oocytes/total number of punctured follicles;

Fertilization rate = number of two pronucleus (PN), 1PN, and 3PN embryos [a spermatozoon enters into a mature oocyte followed by formation of the pronuclei; usually, two pronuclei are seen after fertilization, each containing a haploid set of chromosomes, one set from the oocyte and one from the sperm, before zygote formation (21)]/number of retrieved oocytes (IVF), or number of MII oocytes (ICSI);

2PN cleavage rate =number of 2PN cleavage embryos/number of 2PN embryos;

Available embryo rate = number of available embryos [Good or Fair from D2-D3 cleavage-stage embryos (22); ≥3–5 BC/CB blastocyst (23, 24)]/number of 2PN cleavage embryos;

High-quality embryo rate = number of high-quality [Good from D2-D3 cleavage-stage embryos (22); ≥3–5 BB blastocyst (23, 24)] embryos/number of 2PN cleavage embryos;

Blastocyst formation rate = number of blastocysts from 2PN embryos/number of 2PN cleavage embryos.

### Statistical Analysis

Data analysis was performed using SPSS 23.0 statistical software (Statistical Package for Social Sciences (SPSS), Inc., USA). Quantitative data were expressed as mean ± standard deviation (SD), and classified data were expressed as the rate. The comparison between quantitative data was performed by ANOVA. When multiple groups were compared, the Least Significant Difference (LSD) method was used if the homogeneity of variance was satisfied. If the homogeneity of variance was not satisfied, Dunnett’s T3 method was used. The difference between the classified data groups was analyzed by chi-square test. The Kaplan–Meier curve was used to compare the cumulative pregnancy rates of different groups. p<0.05 was considered to indicate statistically significant differences.

## Results

In all, 1,043 cycles (330 patients) were included in this study. All participants were endometriosis patients with RIF undergoing IVF/ICSI treatment during the period from 2008 to 2018. There were 141 patients (374 cycles) in the untreated group, 94 (326 cycles) in the early-treatment group, and 95 (343 cycles) in the late-treatment group.

Clinical outcomes were compared among three groups. Compared to the untreated group, patients in the treated groups had a higher rate of adenomyosis and ovarian surgery and a lower rate of mild and suspected endometriosis (**Supplemental Table 1**). Proportions of adenomyosis in the early- and late-treatment groups were 12.07% and 7.6%, respectively, which were higher than that of the untreated group (1.09%). In the early group, the proportion of previous ovarian surgery was much higher than the untreated and late-treatment groups (untreated [31.61]% *vs*. early-treatment [13.11%] and late-treatment [19.88%], respectively), and proportion of mild and suspected endometriosis was lower than the untreated and late-treatment groups (untreated [69.54%] *vs*. early-treatment [89.62%] and late-treatment [80.70%], respectively). There was no difference among groups with respect to age, anti-Mullerian hormone antibody (AMH) levels, basic follicle-stimulating hormone (bFSH), and antral follicle count (AFC). The clinical pregnancy rate and live birth rate of the untreated and early- and late-treatment groups showed a gradual increase, while the early abortion rate showed a gradual decline, although this difference was not statistically significant. Strikingly, the treated groups, showed better outcomes in cumulative live birth rates (early-treatment [43.6%] *vs*. late-treatment [46.3%] *vs*. untreated [27.7%], p<0.001, see **Table 1**) even with a history of previous pelvic surgery and adenomyosis.

**Table 1:** Clinical pregnancy outcomes of embryo transfer cycles of endometriosis patients with RIF.

Group Items	The Untreated N = 183	The early treatment N = 174	The late treatment N = 171	P-value
Mean of the previous ET attempts	3.73 ± 0.861	3.94 ± 1.045	4.47 ± 1.428^ab^	**<0.001**
Per capita transfer cycle	1.62 ± 0.99	2.12 ± 0.79	2.01 ± 0.15	
Cycle cancellation rate	23.80% (89/374)	26.69% (87/326)	29.45% (101/343)	0.231
Clinical pregnancy rate	30.05% (55/183)	29.89% (52/174)	32.75% (56/171)	0.811
Early abortion rate	14.55% (8/55)	19.23% (10/52)	12.50% (7/56)	0.612
Live birth rate	21.31% (39/183)	23.56% (41/174)	25.73% (44/171)	0.618
Cumulative live birth rate				
1^st^ cycle	18.4% (26/141)	26.6% (25/94)	30.5% (29/95)	0.086
2^nd^ cycle	24.1% (34/141)	34.0% (32/94)	43.2%^a^ (41/95)	**0.008**
3^rd^ cycle	25.5% (36/141)	40.4%^a^ (38/94)	44.2%^a^ (42/95)	**0.006**
4^th^ cycle	27.7% (39/141)	41.5% (39/94)	45.3%^a^ (43/95)	**0.012**
5^th^ cycle	27.7% (39/141)	42.6% (40/94)	45.3%^a^ (43/95)	**0.010**
Cumulative live birth rate (Total)	27.7% (39/141)	43.6%^a^ (41/94)	46.3%^a^ (44/95)	**0.005**

a P < 0.05, difference was statistically significant when compared to the untreated group.

b P < 0.05, difference was statistically significant when compared to the early-treatment group. All P < 0.05 are highlighted in bold.

When comparing the laboratory outcomes, we found that the adenomyosis rate and ovarian surgery rate of the early-treatment group were the highest (12.79% *vs*. 2.07% *vs*. 4.95%, 39.73% *vs*. 18.67% *vs*. 22.07%, **Supplemental Table 2**). The treated groups, especially the early-treatment group, showed better outcomes than the untreated group, with higher fertilization rates of IVF (early-treated [64.40%] *vs*. late-treated [60.27%] *vs* untreated [46.24%], p<0.001). Although the number was smaller of available (1.75 ± 2.05 *vs*. 2.44 ± 2.83) and high-quality embryos (1.41 ± 1.76 *vs*. 1.80 ± 2.32) in the early-treatment group, the rates of available (90.30% *vs*. 85.20%) and high-quality (76.50% *vs*. 64.47%) embryos seemed much higher than those of the untreated group (**Table 2**).

**Table 2:** Laboratory outcomes of embryo transfer cycles of endometriosis patients with RIF.

Groups Items	The Untreated N=241	The early treatment N=219	The late treated N=222	P-value
Mean of the previous ovum pick up attempts	2.45 ± 1.767	2.96 ± 2.462	3.36 ± 2.589^a^	**0.009**
Per capita egg retrieval cycle	1.78 ± 1.18	2.43 ± 2.34^a^	2.49 ± 2.47^a^	
Oocyte retrieval rate	79.05% (932/1179)	79.56% (545/685)	79.17% (589/744)	0.965
Fertilization rate of IVF	46.24% (431/932)	64.40% (351/545)^a^	60.27% (355/589)^a^	**<0.001**
Fertilization rate of ICSI	80.27% (301/375)	84.07% (95/113)	76.22% (109/143)	0.292
2PN cleavage rate	97.61% (653/669)	98.71% (383/388)	98.28% (400/407)	0.427
Number of available embryos	2.44 ± 2.83	1.75 ± 2.05^a^	1.71 ± 2.11^a^	
Available embryonic rate	85.20% (570/669)	90.30% (363/402)^a^	85.48% (359/420)	**0.042**
Number of high-quality embryos	1.80 ± 2.32	1.41 ± 1.76	1.35 ± 1.89	
High-quality embryonic rate	64.47% (421/653)	76.50% (293/383)^a^	70.75% (283/400)	**<0.001**
Blastocyst formation rate	42.95% (67/156)	45.35% (39/86)	42.86% (45/105)	0.925

a P < 0.05, difference was statistically significant when compared to the untreated group. All P < 0.05 are highlighted in bold.

**Figure 2:**
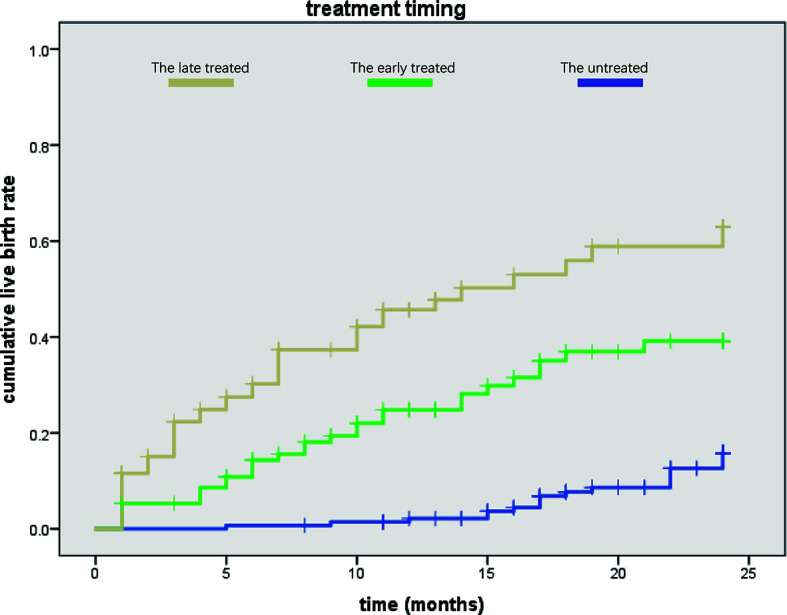
Comparison of the three groups in cumulative live birth rate. The late-treatment group is shown in yellow color, the early-treatment group is shown in green, and the untreated group is shown in blue. We found that the late-treatment group had higher cumulative live birth rate than the other two groups, while the cumulative live birth rate of the untreated group was lowest according to treated time.

We then used Kaplan–Meier curve to analyze the cumulative live birth rate (**Figure 2**). We found that patients in the untreated group needed a mean duration of 23.126 ± 0.252 months to achieve a live birth, while those in the treated groups required a shorter duration (early treatment: 18.479 ± 0.882 months, late treatment: 14.183 ± 1.102 months, **Table 3**).

**Table 3:** The cumulative live birth rate per Kaplan–Meier curves.

Groups	Time to live birth	95%CI	P-value
The untreated	23.126 ± 0.252	[22.602-23.712]	
The early treatment	18.479 ± 0.882	[16.750-20.208]	<0.001
The late treatment	14.183 ± 1.102	[12.023-16.342]	

## Discussion

Endometriosis is a crucial contributor to the decline of female fertility, but thanks to the development of IVF/ICSI technology, a huge population of patients with endometriosis can achieve pregnancy and childbirth.

A historical cohort study (25) recruited 1,027 patients who received IVF/ICSI. After comparing the patients’ pregnancy outcomes between the endometriosis and tubal obstruction groups, it was found that different stages of endometriosis patients have similar outcomes to those with tubal factors after IVF/ICSI. Thus, IVF/ICSI was considered an effective treatment for endometriosis. The question remains whether IVF/ICSI can really solve all infertility-related problems of endometriosis patients and whether it is necessary for endometriosis patients to be pre-treated before IVF/ICSI. In this study, we concluded that even the basic clinical characteristics seemed “worse” in the treatment group, including high rates of adenomyosis and previous ovarian surgery and low rate of mild and suspected endometriosis. Moreover, the pregnancy outcomes of endometriosis patients with RIF were quite improved after clinical treatment. Post treatment, the fertilization rate of IVF and the cumulative live birth rate increased by 14.03–18.16% and 15.17–25.39%, respectively. The available embryonic rate in the early-treatment group was 5% higher than that in the untreated group, while the high-quality embryonic rate was increased by about 11%. The Kaplan–Meier curve analysis also showed that the time spent to achieve live birth in the late- and early-treatment groups was reduced by about 9 months and 5 months, respectively, compared with the untreated group. As endometriosis has an adverse effect on fertility, treatment can benefit endometriosis patients in terms of a successful clinical pregnancy outcome.

Endometriosis can reduce the implantation rate of IVF. A prospective study using oocytes from the same donors which excluded the effects of oocyte and embryo quality, showed that the implantation rate and pregnancy rate of endometriosis patients were significantly lower than those of the control group (26). This may be related to chronic inflammation and endocrine disorders in patients with endometriosis. Chronic aseptic inflammation of the peritoneal cavity, ovary, and uterine cavity of endometriosis patients affects the implantation of embryos (27). In addition, progesterone resistance caused by endometriosis decreases prostaglandin metabolism, increases reactive oxygen species (ROS), activates NFκB-mediated inflammation cascade, and leads to the accumulation of proinflammatory factors, ROS, matrix metalloproteinases (MMPs), and prostaglandins (2). Moreover, endometriosis patients often have genetic aberrations related to cellular production, adhesion, apoptosis, wound healing response, and decidualization (28). All these factors are significant causes of failure of embryo implantation.

Interestingly, the clinical outcomes of the late-treatment group were better than those of the early-treatment group in our study. Although the difference was not statistically significant, the cumulative live birth rate in the late-treatment group exceeded that of the early-treatment group by 3%. The main reasons for this are as follows: First, the time of treatment in the early group was earlier and the diagnosis of RIF required multiple *in vitro* fertilization and embryo transfer/frozen embryo transfer (IVF-ET/FET) cycles, leading to the recurrence of endometriosis which may in turn reduce the chance of fertility. Second, the proportions of unexplained infertility were different between the two treated groups, with the early-treatment group showing a higher proportion than the late-treatment group. Treatment of endometriosis helped many patients with mild or uncomplicated endometriosis to achieve pregnancy before the diagnosis of RIF. Thus, the remaining patients in the early-treatment group were those with severe endometriosis or those complicated with unknown diseases impairing fertility.

Endometriosis can cause a decline in women’s fertility by affecting the quality of oocytes and embryos. A meta-analysis conducted by Senapati (29) of IVF patients with endometriosis and other factors of infertility found that patients with endometriosis had lower fertilization rates. Shebl’s study (30) suggested that patients with endometriosis had lower fertilization rate and more abnormal oocytes than patients in the control group. This may be related to dysregulation of steroids in patients with endometriosis. Ovarian and pelvic endometriosis can reduce the expression of aromatase P450 to impair steroid production in granulosa cells (31) or directly affect the production of steroids, change the cell cycles of granulosa cell, increase their apoptosis, and participate in molecular pathways that alter the development of granulosa cells (32–34). Impaired steroid production may also lead to an imbalance in the secretion of estrogen and progesterone, that affects the maturation of oocytes. In addition, micro environment damage in follicles is another important pathological mechanism of infertility with endometriosis. Oxidative stress is considered a potential factor of endometriosis (35), and ROS are thought to promote meiotic abnormalities and chromatin instability, thereby reducing oocyte quality (36). Because of the treatment of endometriosis, the available and high-quality embryonic rate in the early-treatment group were significantly improved compared with the untreated group. The progesterone resistance and immune disorders in endometriosis patients are alleviated once active endometriosis is controlled. In this way, we can obtain more and better embryos. A meta-analysis by Yang (37) in 2015 showed that the rate of MII oocyte acquisition and the number of embryos formed in untreated ovarian endometriotic cysts group were lower than those in the treated group, which was consistent with our results. However, we found that the early-treatment group had better embryonic outcomes than the late-treatment group, which was somewhat unexpected. In our study, we found the fertilization rate of IVF (64.40% *vs*. 60.27%), available embryonic rate (90.30% *vs*. 85.48%), and high-quality embryonic rate (76.50% *vs*. 70.75%), respectively, were higher in the early- than late-treatment groups (all p>0.05). The possible reason is that patients in late-treatment group had deeper pituitary and ovarian inhibition. It is established that GnRH-a is widely used in endometriosis treatment.

The application of gonadotropins combined with GnRH-a for controlled ovarian stimulation (COS) was first proposed by Porter in 1984. The GnRH-a long protocol is now a popular COS protocol with years of practice (38). However, there is still controversy regarding the dosage of GnRH-a in clinic practice. If GnRH-a is used for a prolonged duration or in large doses, it may inhibit the release of LH surge, reduce ovarian responsiveness, damage the quality of oocytes, affect the function of the corpus luteum, and even affect ovarian hormone secretion (39). Mu et al. (40) also found that excessive application of GnRH-a will affect the number and quality of oocytes and embryos. In our study, although all patients in both treatment groups used a large dose of GnRH-a to control endometriosis activity, the duration from endometriosis treatments to ovarian stimulation of IVF/ICSI was shorter and ovarian suppressed more seriously in the late-treatment group. Thus, patients in the early-treatment group showed a higher rate of fertilization, available embryos, and high-quality embryos than those in the late-treatment group.

Some patients with endometriosis may achieve pregnancy after IVF/ICSI; however, repeated IVF cycles and failure of implantation can case huge psychological and financial burden. Overall, our results showed that the treatment of endometriosis was necessary. Although the use of GnRH-a may inhibit ovarian function and affect the quality of oocytes and embryos, patients with endometriosis could still have good live birth outcomes after treatment. We explored the relationship between endometriosis and RIF as well as the importance of endometriosis treatment to provide a theoretical basis for clinical applications which may optimize assisted reproduction strategies to help patients achieve better pregnancy outcomes.

However, we have not compared the specific treatment protocols and effectiveness of such protocols for endometriosis patients in this study which require further detailed investigation in the future.

In conclusion, it is necessary to identify and control the activity of endometriosis so that endometriosis patients may benefit from and achieve successful clinical outcomes with IVF/ICSI treatments.

## Data Availability Statement

The raw data supporting the conclusions of this article will be made available by the authors, without undue reservation.

## Ethics Statement

The studies involving human participants were reviewed and approved by Ethics Committee of Jiangsu Province Hospital. The patients/participants provided their written informed consent to participate in this study.

## Author Contributions

YM conceived the study, including the procedures, conception, and design. CZ, LG, LS, and ZH were responsible for data collection. CZ contributed to the data analysis and drafted the article. LC and JH participated in the interpretation of data. JL revised the article. All authors contributed to the article and approved the submitted version.

## Funding

This work was supported by the National Key Research and Development Program of China (2017YFC1001604, 2016YFC1000603); the National Natural Science Foundation of China (81671438, 81701517); and a program of Maternal and Child Health of Jiangsu Province China (FYX201917).

## Conflict of Interest

The authors declare that the research was conducted in the absence of any commercial or financial relationships that could be construed as a potential conflict of interest.

## Publisher’s Note

All claims expressed in this article are solely those of the authors and do not necessarily represent those of their affiliated organizations, or those of the publisher, the editors and the reviewers. Any product that may be evaluated in this article, or claim that may be made by its manufacturer, is not guaranteed or endorsed by the publisher.

## References

[1] Vander Borght M Wyns C. Fertility and Infertility: Definition and Epidemiology. Clin Biochem (2018) 62:2–10. 10.1016/j.clinbiochem.2018.03.012 29555319

[2] Lin YH Chen YH Chang HY Au HK Tzeng CR Huang YH. Chronic Niche Inflammation in Endometriosis-Associated Infertility: Current Understanding and Future Therapeutic Strategies. Int J Mol Sci (2018) 19(8):2385. 10.3390/ijms19082385 PMC6121292 30104541

[3] Miller JE Ahn SH Monsanto SP Khalaj K Koti M Tayade C. Implications of Immune Dysfunction on Endometriosis Associated Infertility. Oncotarget (2017) 8(4):7138–47. 10.18632/oncotarget.12577 PMC5351695 27740937

[4] Sanchez AM Vanni vs Bartiromo L Papaleo E Zilberberg E Candiani M. Is the Oocyte Quality Affected by Endometriosis? A Review of the Literature. J Ovarian Res (2017) 10(1):43. 10.1186/s13048-017-0341-4 28701212 PMC5508680

[5] Coccia ME Rizzello F Mariani G Bulletti C Palagiano A Scarselli G. Impact of Endometriosis on *In Vitro* Fertilization and Embryo Transfer Cycles in Young Women: A Stage-Dependent Interference. Acta Obstet Gynecol Scand (2011) 90(11):1232–8. 10.1111/j.1600-0412.2011.01247.x 21793811

[6] Al-Azemi M Bernal AL Steele J Gramsbergen I Barlow D Kennedy S. Ovarian Response to Repeated Controlled Stimulation in *In-Vitro* Fertilization Cycles in Patients With Ovarian Endometriosis. Hum Reprod (2000) 15(1):72–5. 10.1093/humrep/15.1.72 10611191

[7] Boucret L Bouet P-E Riou J Legendre G Delbos L Hachem HE. Endometriosis Lowers the Cumulative Live Birth Rates in IVF by Decreasing the Number of Embryos But Not Their Quality. J Clin Med (2020) 9(8):2478. 10.3390/jcm9082478 PMC7464781 32752267

[8] Ledee N Petitbarat M Chevrier L Vitoux D Vezmar K Rahmati M. The Uterine Immune Profile May Help Women With Repeated Unexplained Embryo Implantation Failure After *In Vitro* Fertilization. Am J Reprod Immunol (2016) 75(3):388–401. 10.1111/aji.12483 26777262 PMC4849202

[9] Demirol A Gurgan T. Effect of Treatment of Intrauterine Pathologies With Office Hysteroscopy in Patients With Recurrent IVF Failure. Reprod BioMed Online (2004) 8(5):590–4. 10.1016/S1472-6483(10)61108-X 15151729

[10] Orvieto R Brengauz M Feldman B. A Novel Approach to Normal Responder Patient With Repeated Implantation Failures–A Case Report. Gynecol Endocrinol: Off J Int Soc Gynecol Endocrinol (2015) 31(6):435–7. 10.3109/09513590.2015.1005595 25731193

[11] Polanski LT Baumgarten MN Quenby S Brosens J Campbell BK Raine-Fenning NJ. What Exactly Do We Mean by ‘Recurrent Implantation Failure’? A Systematic Review and Opinion. Reprod BioMed Online (2014) 28(4):409–23. 10.1016/j.rbmo.2013.12.006 24581986

[12] Coughlan C Ledger W Wang Q Liu F Demirol A Gurgan T. Recurrent Implantation Failure: Definition and Management. Reprod BioMed Online (2014) 28(1):14–38. 10.1016/j.rbmo.2013.08.011 24269084

[13] Bashiri A Halper KI Orvieto R. Recurrent Implantation Failure-Update Overview on Etiology, Diagnosis, Treatment and Future Directions. Reprod Biol Endocrinol (2018) 16(1):121. 10.1186/s12958-018-0414-2 30518389 PMC6282265

[14] Kim JW Shim SH Lee WS. De Novo Balanced Reciprocal Translocation T(2;3)(Q31;Q27) in a Fetus Conceived Using PGD in a T(2;14)(Q35;Q32.1) Balanced Reciprocal Translocation Carrier Mother. Clin Case Rep (2017) 5(6):841–4. 10.1002/ccr3.932 PMC5457991 28588822

[15] Inagaki N Stern C McBain J Lopata A Kornman L Wilkinson D. Analysis of Intra-Uterine Cytokine Concentration and Matrix-Metalloproteinase Activity in Women With Recurrent Failed Embryo Transfer. Hum Reprod (2003) 18(3):608–15. 10.1093/humrep/deg139 12615834

[16] Santillan I Lozano I Illan J Verdu V Coca S Bajo-Arenas JM. Where and When Should Natural Killer Cells be Tested in Women With Repeated Implantation Failure? J Reprod Immunol (2015) 108:142–8. 10.1016/j.jri.2014.12.009 25708533

[17] Zhao J Zhang Q Wang Y Li Y. Endometrial Pattern, Thickness and Growth in Predicting Pregnancy Outcome Following 3319 IVF Cycle. Reprod BioMed Online (2014) 29(3):291–8. 10.1016/j.rbmo.2014.05.011 25070912

[18] Kasius A Smit JG Torrance HL Eijkemans MJ Mol BW Opmeer BC. Endometrial Thickness and Pregnancy Rates After IVF: A Systematic Review and Meta-Analysis. Hum Reprod Update (2014) 20(4):530–41. 10.1093/humupd/dmu011 24664156

[19] Exacoustos C Zupi E Piccione E. Ultrasound Imaging for Ovarian and Deep Infiltrating Endometriosis. Semin Reprod Med (2017) 35(1):5–24. 10.1055/s-0036-1597127 28076877

[20] Nisenblat V Bossuyt PMM Shaikh R Farquhar C Jordan V Scheffers CS. Blood Biomarkers for the non-Invasive Diagnosis of Endometriosis. Cochrane Database System Rev (2016) 5):CD012179. 10.1002/14651858.CD012179 PMC7076288 27132058

[21] Zegers-Hochschild F Adamson GD Dyer S Racowsky C de Mouzon J Sokol R. The International Glossary on Infertility and Fertility Care, 2017. Fertil Steril (2017) 108(3):393–406. 10.1016/j.fertnstert.2017.06.005 28760517

[22] Yang ST Shi JX Gong F Zhang SP Lu CF Tan K. Cleavage Pattern Predicts Developmental Potential of Day 3 Human Embryos Produced by IVF. Reprod Biomedicine Online (2015) 30(6):625–34. 10.1016/j.rbmo.2015.02.008 25892500

[23] Men CJ Bormann CL Walsh BW Racowsky C. Is the Presence of an Uncleaved Embryo on Day 3 a Useful Predictor of Outcomes Following Day 5 Transfer? J Assisted Reprod Genet (2015) 32(9):1379–84. 10.1007/s10815-015-0532-0 PMC4595402 26206457

[24] Gardner DK Schoolcraft WB. *In Vitro* Culture of Human Blastocysts. In: Jansen R, editor. Towards Reprod Certainty Infertility Genet Beyond. Carnforth, NY: Parthenon Publishing (1999). p. 378–88.

[25] Dong X Liao X Wang R Zhang H. The Impact of Endometriosis on IVF/ICSI Outcomes. Int J Clin Exp Pathol (2013) 6(9):1911–8. 10.1155/2013/416870 PMC3759500 24040458

[26] Prapas Y Goudakou M Matalliotakis I Kalogeraki A Matalliotaki C Panagiotidis Y. History of Endometriosis may Adversely Affect the Outcome in Menopausal Recipients of Sibling Oocytes. Reprod BioMed Online (2012) 25(5):543–8. 10.1016/j.rbmo.2012.07.020 23000083

[27] Lessey BA Kim JJ. Endometrial Receptivity in the Eutopic Endometrium of Women With Endometriosis: It is Affected, and Let Me Show You Why. Fertil Steril (2017) 108(1):19–27. 10.1016/j.fertnstert.2017.05.031 28602477 PMC5629018

[28] Ahn SH Khalaj K Young SL Lessey BA Koti M Tayade C. Immune-Inflammation Gene Signatures in Endometriosis Patients. Fertil Steril (2016) 106(6):1420–31.e7. 10.1016/j.fertnstert.2016.07.005 27475412 PMC5683404

[29] Senapati S Sammel MD Morse C Barnhart KT. Impact of Endometriosis on *In Vitro* Fertilization Outcomes: An Evaluation of the Society for Assisted Reproductive Technologies Database. Fertil Steril (2016) 106(1):164–71.e1. 10.1016/j.fertnstert.2016.03.037 27060727 PMC5173290

[30] Shebl O Sifferlinger I Habelsberger A Oppelt P Mayer RB Petek E. Oocyte Competence in *In Vitro* Fertilization and Intracytoplasmic Sperm Injection Patients Suffering From Endometriosis and its Possible Association With Subsequent Treatment Outcome: A Matched Case-Control Study. Acta Obstet Gynecol Scand (2017) 96(6):736–44. 10.1111/aogs.12941 27317364

[31] Sanchez AM Somigliana E Vercellini P Pagliardini L Candiani M Vigano P. Endometriosis as a Detrimental Condition for Granulosa Cell Steroidogenesis and Development: From Molecular Alterations to Clinical Impact. J Steroid Biochem Mol Biol (2016) 155(Pt A):35–46. 10.1016/j.jsbmb.2015.07.023 26407755

[32] Toya M Saito H Ohta N Saito T Kaneko T Hiroi M. Moderate and Severe Endometriosis Is Associated With Alterations in the Cell Cycle of Granulosa Cells in Patients Undergoing *In Vitro* Fertilization and Embryo Transfer. Fertil Steril (2000) 73(2):344–50. 10.1016/S0015-0282(99)00507-5 10685541

[33] Sanchez AM Vigano P Quattrone F Pagliardini L Papaleo E Candiani M. The WNT/Beta-Catenin Signaling Pathway and Expression of Survival Promoting Genes in Luteinized Granulosa Cells: Endometriosis as a Paradigm for a Dysregulated Apoptosis Pathway. Fertil Steril (2014) 101(6):1688–96. 10.1016/j.fertnstert.2014.02.040 24661731

[34] Sanchez AM Vigano P Somigliana E Panina-Bordignon P Vercellini P Candiani M. The Distinguishing Cellular and Molecular Features of the Endometriotic Ovarian Cyst: From Pathophysiology to the Potential Endometrioma-Mediated Damage to the Ovary. Hum Reprod Update (2014) 20(2):217–30. 10.1093/humupd/dmt053 24129684

[35] Carvalho LF Abrao MS Biscotti C Sharma R Nutter B Falcone T. Oxidative Cell Injury as a Predictor of Endometriosis Progression. Reprod Sci (2013) 20(6):688–98. 10.1177/1933719112466301 23287096

[36] Mansour G Sharma RK Agarwal A Falcone T. Endometriosis-Induced Alterations in Mouse Metaphase II Oocyte Microtubules and Chromosomal Alignment: A Possible Cause of Infertility. Fertil Steril (2010) 94(5):1894–9. 10.1016/j.fertnstert.2009.09.043 19896655

[37] Yang C Geng Y Li Y Chen C Gao Y. Impact of Ovarian Endometrioma on Ovarian Responsiveness and IVF: A Systematic Review and Meta-Analysis. Reprod BioMed Online (2015) 31(1):9–19. 10.1016/j.rbmo.2015.03.005 25982092

[38] Porter RN Smith W Craft IL Abdulwahid NA Jacobs HS. Induction of Ovulation for *In-Vitro* Fertilisation Using Buserelin and Gonadotropins. Lancet (1984) 324(8414):1284–5. 10.1016/S0140-6736(84)92840-X 6150318

[39] Brus L Lambalk CB de Koning J Helder MN Janssens RM Schoemaker J. Specific Gonadotrophin-Releasing Hormone Analogue Binding Predominantly in Human Luteinized Follicular Aspirates and Not in Human Pre-Ovulatory Follicles. Hum Reprod (Oxford England) (1997) 12(4):769–73. 10.1093/humrep/12.4.769 9159440

[40] Mu Z-N Sun Z-G Song J-Y Liu H-G Qiao Y Xia Q-C. Effect of Duration of Gonadotropin Releasing Hormone Agonist on the Outcome of *In Vitro* Fertilization-Embryo Transfer in a Short-Acting Long Regimen. Libyan J Med (2019) 14(1):1652058. 10.1080/19932820.2019.1652058 31405338 PMC8896834

