# Supplementary figures and images for: Analysis of IVF/ICSI Outcomes in Endometriosis Patients With Recurrent Implantation Failure: Influence on Cumulative Live Birth Rate
Source: Front Endocrinol (Lausanne). 2021 Jul 30;12:640288. doi: 10.3389/fendo.2021.640288 (PMC8362597; doi:10.3389/fendo.2021.640288)

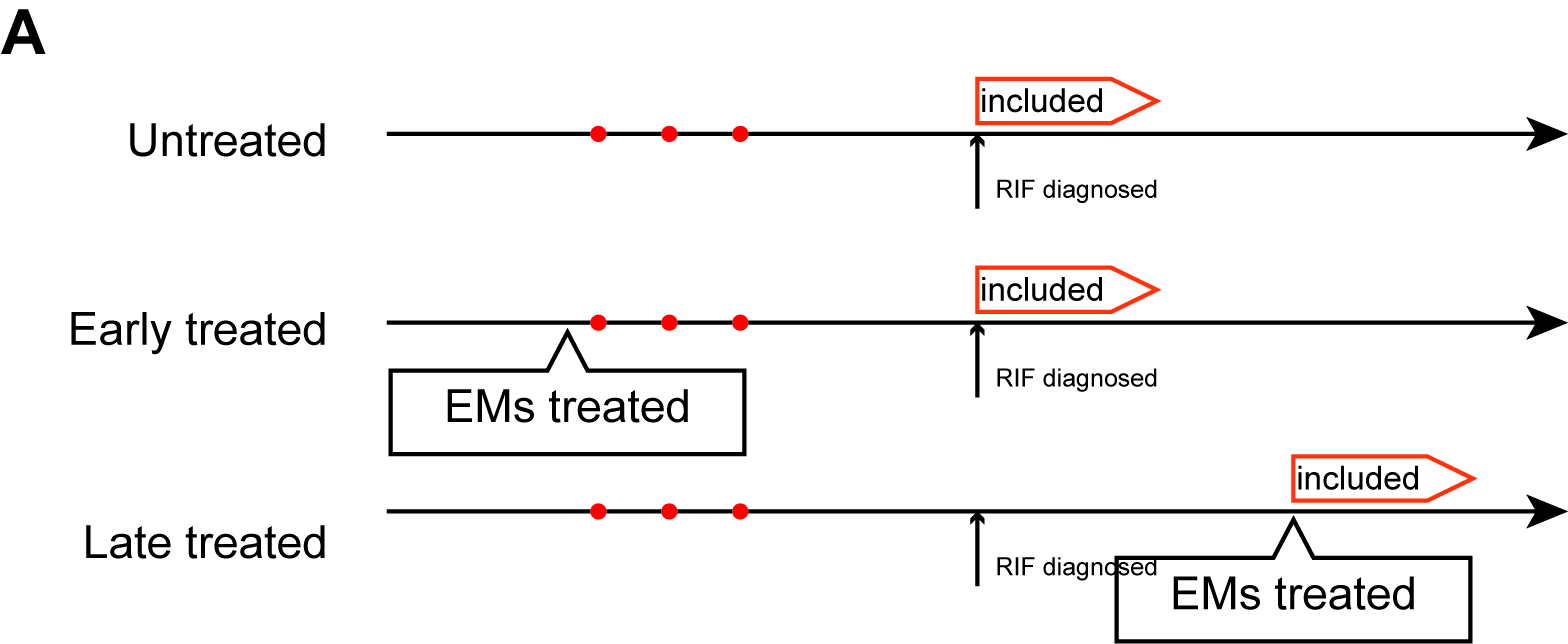

Supplement: Supplementary file 1 [file Image_1.tif]
