# Supplementary material for: Analysis of IVF/ICSI Outcomes in Endometriosis Patients With Recurrent Implantation Failure: Influence on Cumulative Live Birth Rate
Source: Front Endocrinol (Lausanne). 2021 Jul 30;12:640288. doi: 10.3389/fendo.2021.640288 (PMC8362597; doi:10.3389/fendo.2021.640288)
Supplement: Supplementary file 2 [file Table_1.docx]

**Supplemental table 1** General situations of embryo transfer cycles of EMs patients with RIF

| Groups  Items | Embryo transfer cycles | | | |
| --- | --- | --- | --- | --- |
|  | The untreated | The early treated | The late treated | P-value |
| Cycles | 183 | 174 | 171 |  |
| Primary/secondary infertility | 101/82 | 74/100 | 114/57 |  |
| Ages | 34.87±4.65 | 34.36±4.17 | 35.39±4.62 | 0.102 |
| Infertility duration | 6.58±4.03 | 7.03±3.86 | 7.47±3.94 | 0.107 |
| BMI (kg/m^2)^ | 22.03±2.52 | 21.99±2.17 | 21.73±2.18 | 0.419 |
| AMH (ng/ml) | 5.03±4.31 | 4.54±3.81 | 4.38±3.67 | 0.608 |
| bFSH (IU/L) | 7.80±2.13 | 8.31±2.26 | 8.29±2.83 |  |
| AFC | 10.17±5.94 | 10.45±6.27 | 11.18±5.63 | 0.264 |
| Number of embryos transferred per cycle | 1.43±0.50 | 1.29±0.45 | 1.33±0.48 |  |
| Blastocyst transfer rate (%) | 19.67% (36/183) | 16.09% (28/174) | 18.71% (32/171) | 0.665 |
| Adenomyosis rate (%) | 1.09% (2/183) | 12.07% (21/174) ^a^ | 7.60% (13/171) ^a^ | **＜0.001** |
| Ovarian surgery rate (%) | 13.11% (24/183) | 31.61% (55/174) ^a^ | 19.88% (34/171) ^b^ | **＜0.001** |
| Mild and suspected EMs rate (%) | 89.62% (164/183) | 69.54% (121/174) ^a^ | 80.70% (138/171) ^b^ | **＜0.001** |
| Down-regulated cycles rate (%) | 27.32% (50/183) | 27.01% (47/174) | 29.82% (51/171) | 0.816 |
| Artificial menstrual cycles rate (%) | 15.85% (29/183) | 13.22% (23/174) | 15.79% (27/171) | 0.733 |
| Natural/ Mild-stimulation cycles rate (%) | 54.64% (100/183) | 60.34% (105/174) | 58.48% (100/171) | 0.538 |

^a^ P＜0.05, difference was statistically significant when compared to group A,

^b^ P＜0.05, difference was statistically significant when compared to group B
