# Supplementary material for: Analysis of IVF/ICSI Outcomes in Endometriosis Patients With Recurrent Implantation Failure: Influence on Cumulative Live Birth Rate
Source: Front Endocrinol (Lausanne). 2021 Jul 30;12:640288. doi: 10.3389/fendo.2021.640288 (PMC8362597; doi:10.3389/fendo.2021.640288)
Supplement: Supplementary file 3 [file Table_2.docx]

**Supplemental table 2** General situations of embryo retrieval cycles of EMs patients with RIF

| groups  items | Ovum pick up | | | | | | | P-value |
| --- | --- | --- | --- | --- | --- | --- | --- | --- |
|  | The Untreated | | The early treated | | | The late treated | |  |
| Cycles | 241 | 219 | | | 222 | | |  |
| Primary/secondary infertility | 131/110 | 91/128 | | | 145/77 | | |  |
| Ages | 35.42±5.00 | 34.78±4.09 | | | 35.68±4.57 | | |  |
| Infertility duration | 6.50±4.10 | 6.91±3.49 | | | 7.77±4.19^a^ | | |  |
| BMI (kg/m^2)^ | 22.05±2.63 | 21.92±2.18 | | | 21.63±2.18 | | |  |
| AMH (ng/ml) | 4.19±4.11 | 3.57±3.40 | | | 4.09±3.50 | | |  |
| bFSH (IU/L) | 8.51±4.14 | 8.70±2.50 | | | 8.58±2.76 | | | 0.830 |
| AFC | 9.53±6.07 | 9.05±5.88 | | | 9.27±5.72 | | | 0.088 |
| Adenomyosis rate (%) | 2.07% (5/241) | 12.79% (28/219) ^a^ | | | 4.95% (11/222) ^b^ | | | **＜0.001** |
| Ovarian surgery rate (%) | 18.67% (45/241) | 39.73% (87/219) ^a^ | | | 22.07% (49/222) ^b^ | | | **＜0.001** |
| Mild and suspected EMs rate (%) | 80.08% (193/241) | 59.82% (131/219) ^a^ | | | 81.53% (181/222) ^b^ | | | **＜0.001** |
| Down-regulated cycles rate (%) | 10.79% (26/241) | | | 10.50% (23/219) | | | 17.57% (39/222) | 0.041 |
| Natural/ Mild-stimulation cycles rate (%) | 30.29% (73/241) | | | 40.64% (89/219) | | | 35.59% (79/222) | 0.068 |

^a^ P＜0.05, difference was statistically significant when compared to group A,

^b^ P＜0.05, difference was statistically significant when compared to group B
